# The clinical outcome of total knee arthroplasty is compromised by a previously implanted medial unicondylar knee arthroplasty

**DOI:** 10.1007/s00402-023-04829-7

**Published:** 2023-03-18

**Authors:** M. C. Liebensteiner, A. Ruzicka, M. Hinz, H. Leitner, A. Harrasser, D. Dammerer, M. Krismer

**Affiliations:** 1 Department for Orthopaedic Surgery, Medical University Innsbruck, Innsbruck, Austria; 2 Department of Orthopaedic Sports Medicine, Technical University of Munich, Munich, Germany; 3 Department of Clinical Epidemiology, Tyrolean Federal Institute for Integrated Care, Tirol Kliniken GmbH, Innsbruck, Austria; 4 Department of Orthopaedics and Traumatology, University Hospital Krems, Krems, Austria

**Keywords:** Orthopedic surgery, Revision, Revision arthroplasty, Conversion arthroplasty, TKA, UKA

## Abstract

**Objective:**

To investigate the clinical outcome of patients that underwent conversion of a medial unicondylar knee arthroplasty (UKA) to a total knee arthroplasty (TKA) and to compare that outcome to patients that underwent primary TKA. It was hypothesized that those groups would significantly differ in terms of knee score outcome and implant survival.

**Methods:**

A retrospective-comparative study was conducted utilizing data from the Federal state’s arthroplasty registry. Included were patients from our department that undergone a conversion of a medial UKA to a TKA (UKA-TKA group). The Western Ontario and MacMaster Universities Osteoarthritis Index (WOMAC) from preoperative and 1-year postoperative was used. Moreover, the implant survival was analyzed.

**Results:**

In the UKA-TKA group, there were 51 cases (age 67 ± 10, 74% women), and in the TKA group, there were 2247 cases (age 69 ± 9, 66% women). The one-year postoperative WOMAC total score was 33 in the UKA-TKA group und 21 in the TKA group (*p* < 0.001). Similarly, the WOMAC pain, WOMAC stiffness, and WOMAC function scores were significantly worse in the UKA-TKA. After 5 years, the survival rates were 82% and 95% (*p* = 0.001). The 10-years prosthesis survival was 74% and 91% in the UKA-TKA and TKA groups, respectively (*p* < 0.001).

**Conclusions:**

Based on our findings it is concluded that patients who received a TKA after UKA have inferior results than those that directly receive a TKA. This is true for both patient-reported knee outcome and prosthesis survival. Converting UKA to TKA should not be seen as an easy operation, but should rather be done by surgeons with considerable experience in both primary and revision knee arthroplasty.

## Introduction

It was reported that early failure modes for medial unicondylar knee arthroplasty (UKA) were mainly bearing dislocation, fracture, and infection, whereas the major late failure mode was progression of osteoarthritis [1], disregarding the type of UKA: cemented, uncemented or hybrid [2]. Regardless of the reasons for revision, when a UKA has to be revised to a total knee arthroplasty (TKA), it is a common discussion in the orthopedic community whether the outcome of total knee arthroplasty (TKA) is compromised by a previously implanted medial UKA.

A comprehensive literature analysis was carried out with this regard. Overall 29 original articles dealing with the subject of converting UKA to TKA were identified [3, 4, 6–31, 34] (Table 1).

**Table 1:** Comprehensive Literature review regarding publications dealing with unicondylar knee arthroplasty converted to total knee arthroplasty (UKA-TKA)

Author	Year	Objective	Cases	LoE	Implant UKA	Implant TKA	Outcome parameter	Results
Barrett	1987	UKA-TKA	29	4	Not reported	Not reported	HSS, radiographs	66%: good/excellent outcome
*Becker*	*2004*	*UKA-TKA vs TKA*	*28 vs. 28*	*3*	*Divers*	*Natural Knee (Sulzer)*	*KSS; WOMAC, radiographic outcome, ROM, poly size*	*UKA-TKA: thicker poly, worse ROM, worse knee scores (KSS function and WOMAC function)*
Berend	2009	UKA-TKA	50	4	Divers	divers	Differences between types of UKA being revised	All poly UKA more difficut to revise
Chakrabarty	1998	UKA-TKA	53	4	Divers	Divers	Bristol Knee Score	79%; Excellent/good score outcome
Chou	2012	UKA-TKA	33	4	Divers	Divers	OKS	OKS 1 y postop: 29
Dudley	2008	UKA-TKA vs TKA-TKA	68 vs. 112	3	Divers	Divers	Costs, operating time, survival, bone loss	UKA-TKA: lower costs, less bone loss (poly size), less operation time, no diff in survival
Gill	1995	UKA-TKA vs HTO-TKA	30 vs. 30	3	Not reported	Divers	KSS	KSS better in HTO- TKA
Hang	2010	UKA-TKA vs TKA-TKA	Not reported	3	Divers	Divers	CRR	5y CRR UKA-TKA: 15%, TKA-TKA:18%
Jackson	1994	UKA-TKA vs HTO-TKA	20 vs. 23	3	ST. Georg (LINK)	Divers	Complications, knee scores, ROM	Scores and ROM similar, HTO-TKA: more complications (wound healing), UKA-TKA: more problems with bone loss
*Järvenpää*	*2010*	*UKA-TKA vs TKA*	*21 vs. 28*	*3*	*Not reported*	*Divers*	*Complications, ROM, WOMAC, VAS pain, walking distance, get up and go test*	*UKA-TKA: signif. worse WOMAC pain and stiffness (no other differences)*
Johnson	2007	UKA-TKA	77	4	Divers	Divers	Survival, Bristol Knee Score	10y survival: 91%, Bristol Score 78
Kerens	2013	UKA-TKA	30	3	Divers	Divers	OKS, VAS pain	UKA-TKA with precise Diagnosis: signific. better outcome
Kerens	2013	UKA-TKAwith PSI	10	4	Oxford (Biomet)	Vanguard (Biomet)	HKA, component positioning	3 of 10 were radiographic outliers (± 3°)
Lai	1993	UKA-TKA	48	4	Divers	Divers	HSS, KSS	Scores improved signif
Levine	1996	UKA-TKA	31	4	Brigham (J & J)	Divers	KSS, KSS radiographic system, ROM	KSS 91/81, ROM 115
Lewold	1998	UKA-TKA vs. UKA-UKA	750 vs 232	3	Divers	Divers	CRR	5y CRR:7% vs 26%
Martin	1995	UKA-TKA	23	4	Oxford (Biomet)	Divers	KSS, KSS radiographic system, ROM	KSS: 10 excellent, 3 good
McAuley	2001	UKA-TKA	32	4	Divers	Divers	KSS; ROM, complications	KSS 89/81, ROM: 111°,
*Miller*	*2002*	*UKA-TKA vs TKA*	*35 vs 100*	*3*	*Divers*	*Not reported*	*KSS, complications*	*UKA-TKA: less KSS gain pre-postop*
*O’Donnell*	*2013*	*UKA-TKA vs TKA*	*55 vs 55*	*3*	*Divers*	*divers*	*ROM, KSS,radiographs*	*No differences*
Oduwole	2010	UKA-TKA	14	4	Oxford	divers	WOMAC, SF-36	No improvement in scores
Otte	1997	UKA-TKA	29	4	Divers	AGC (Biomet)	HSS, radiographs	Excellent/ Good: 69%
Padgett	1991	UKA-TKA	19	4	Divers	Divers	HSS, KSS radiograhic analysis	UKA-TKA: results satisfactory (but not good), similar results to Revision TKA
*Pearse*	*2010*	*UKA-TKA vs TKA*	*122 vs.13257*	*3*	*Divers*	*Divers*	*CRR, OKS 6mopostop*	*UKA-TKA: signif. higher CRR than TKA, and signif. worse OKS*
Saldanha	2007	UKA-TKA	36	4	Oxford (Biomet)	Divers	KSS, radiographs	KSS knee 86, KSS function 78
Saragaglia	2009	UKA-TKA	27	4	Divers	Divers	KSS, ROM	KSS 86 / 80; ROM: 104°
*Sarraf*	*2013*	*UKA-TKA vs TKA*	*374 vs251803*	*3*	*Divers*	*Divers*	*Poly-Size (= Bone Loss), Constraint Level*	*PE size: TKA 10 mm, UKA-TKA 12.79 mm. Constraint: TKA 2.15%; UKA-TKA 4.19%*
Springer	2006	UKA-TKA	22	4	Divers	Divers	KSS, ROM	Signif. improved KSS, ROM unchanged
Wynn Jones	2012	UKA-TKA	80	4	Oxford (Biomet)	Divers	OKS, SF-12	OKS: 32, SF-12: 31

Those comparing UKA-TKA with TKA are marked in italic

*LoE* level of evidence, *UKA* unicondylar knee arthroplasty, *TKA* total knee arthroplasty, *UKA-TKA* UKA converted to TKA, *HSS* Hospital for Special Surgery Score, *WOMAC* Western Ontario and McMaster Universities Osteoarthritis Index, *ROM* range of motion, *KSS* Knee Society Score, *OKS* Oxford Knee Score, *HTO* high tibial osteotomy, *TKA-TKA* TKA converted to TKA, *CRR* cumulative revision rate, *HTO-TKA* HTO converted to TKA, *VAS* visual analog scale, *SF-12* short-form 12, *SF-36* short-form 36

Interestingly, the majority of those studies only reported non-controlled case series of patients who’s UKA were converted to TKA. Only 6 studies compared patients with UKA converted to TKA (UKA-TKA) with patients that received primary TKA [4, 13, 22, 23, 27, 30]. Regarding implant survival (or cumulative revision rate) and patient-reported outcome (PRO) like knee scores as strongest possible outcome parameters, it seems that neither of those studies applied both. Moreover, some of those six publications suffered from rather low sample sizes (e.g., 21 vs. 28 [13]) and others followed their patients only over relatively short periods (e.g., 4 years [2]). Overall, only one previous research group analyzed the cumulative revision rate of UKA-TKA compared to TKA [27].

In summary, the majority of publications were case series of UKA to TKA conversions (Level of Evidence 4). Those studies that compared UKA-TKA to TKA (Level of Evidence 3) suffered from the above-mentioned limitations.

Consequently, it was the aim of the current study to investigate the clinical outcome of patients that underwent conversion of a medial UKA to a TKA (UKA-TKA group). And to compare that outcome to patients that underwent primary TKA (TKA group). It was hypothesized that those groups would significantly differ in terms of knee score outcome (H1) and implant survival (H2).

## Methods

The study design was retrospective-comparative. Data from the arthroplasty registry was utilized after approval by the ethics committee of the Medical University (approval No. AN2016-0207). Patients who previously underwent either primary TKA (TKA group) or conversion of a medial UKA to a TKA (UKA-TKA group) at our department were considered. Cases were excluded in the case of incomplete WOMAC data. Among those patients in the registry databank with primary TKA, those with revision implants (very complex primaries) or tumor prosthesis were excluded, therefore leaving only cruciate retaining (CR) and posterior stabilized (PS) implants for the TKA group.

For patient-reported outcome measurement, the Western Ontario and MacMaster Universities Osteoarthritis Index (WOMAC) score [5] was available from the arthroplasty registry. It had been applied in the German language version [32] the day before surgery and again postoperatively 1 year after surgery.

The WOMAC questionnaire collects data on pain, stiffness, and physical function. Every item was completed on an 11-point scale and converted for analysis purposes to a scale from 0 to 100%, 0 denoting the best and 100% the worst response. The score for each of the three main dimensions is defined as the sum of all item scores divided by the number of items. The total score was defined as the sum of pain, stiffness, and function scores divided by three. Prosthesis survival data was also taken from the arthroplasty registry data bank.

For statistical analysis, Stata 13 (StataCorp. 2013. Stata Statistical Software: Release 13. College Station, TX: StataCorp LP) was used. Means and standard deviations were calculated as descriptives. Independent *T*-Tests were applied to test for differences in WOMAC scores between the groups. Statistical tests were always performed two-tailed. Alpha was defined as 0.05. We estimated cumulative revision-free survival from date of surgery until date of revision, date of death or end of follow-up, whichever occurred first, by applying the Kaplan–Meier method. Differences in survival curves were tested using the generalized Fleming-Harrington test of equality, with parameters *q* and *p* chosen at *p* = 0.0, *q* = 0.03.

## Results

In the UKA-TKA group, there were 51 cases (age 67 ± 10, 74% women), and in the TKA group, there were 2247 cases (age 69 ± 9, 66% women). For the UKA-TKA group, the reasons for revision are provided in Table 2.

**Table 2:** Reasons (frequencies) for converting medial unicondylar knee arthroplasty (UKA) to total knee arthroplasty (TKA)

	Numbers
Unexplained pain	10
Aseptic loosening	20
Progression of osteoarthritis	15
Valgus deformity	3
Bearing dislocation	1
Instability	1
Wear	1
Total	51

In 22 cases, a cruciate retaining design was used (Stryker, Scorpio, and Triathlon CR). In 27 cases, a posterior stabilized design was used (Stryker, Scorpio, and Triathlon PS). One case needed a semi-constrained (Triathlon TS) and one case a constrained implant design (Link, Rotating Hinge, Endo Model). In the primary TKA group, the implants used were Scorpio CR, Scorpio PS, Triathlon CR, and Triathlon PS. The 1-year postoperative WOMAC total score was 33 in the UKA-TKA group and 21 in the TKA group (*p* < 0.001). Similarly, the WOMAC pain, WOMAC stiffness, and WOMAC function scores were significantly worse in the UKA-TKA group one year postoperatively (0.001 < *p* <  0.007, H1, Table 3).

**Table 3:** WOMAC Outcome for the two groups with respective *p* values

	UKA-TKA	TKA	*p* value
WOMAC total 1y	33 ± 21	21 ± 20	< 0.001
WOMAC pain 1y	28 ± 21	17 ± 20	0.001
WOMAC stiffness 1y	39 ± 26	24 ± 23	< 0.001
WOMAC function 1y	32 ± 22	21 ± 20	0.007

Provided are means and standard deviations

*UKA-TKA* unicondylar knee arthroplasty converted to total knee arthroplasty, *TKA* total knee arthroplasty, *1y* 1 Year postoperative, *WOMAC* Western Ontario and MacMaster Universities Osteoarthritis Index

**Fig. 1:**
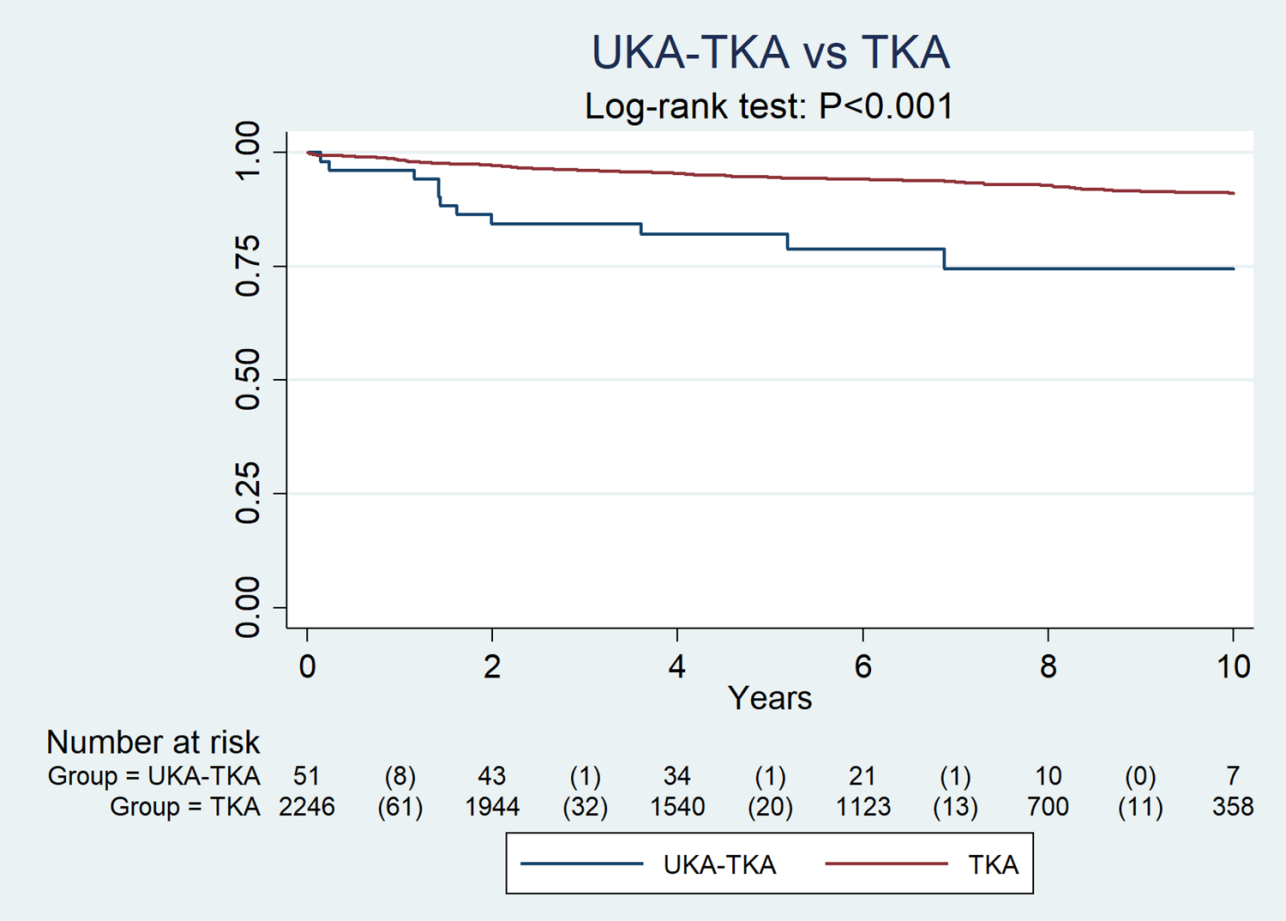
Implant survival for unicondylar knee arthroplasty converted to total knee arthroplasty (UKA-TKA group) and for primary total knee arthroplasty (TKA)

The 3-year prosthesis survival was 84% and 96% in the UKA-TKA and TKA groups, respectively. After 5 years, the survival rates were 82% and 95%. The 10-year prosthesis survival was 74% and 91% in the UKA-TKA and TKA groups, respectively (*p* < 0.001, H2, Table 4, Fig. 1).

**Table 4:** Survival for UKA-TKA group and TKA group

	Survival	Standard-error
UKA-TKA (year)		
1	0.9608	0.0272
3	0.8431	0.0509
5	0.8204	0.0544
10	0.7438	0.0719
TKA (year)		
1	0.9827	0.0028
3	0.9613	0.0042
5	0.9454	0.0052
10	0.9096	0.0084

## Discussion

Regarding the hypotheses of the study, the most important findings were that (a) UKA-TKA provided significantly worse WOMAC scores than TKA and (b) that UKA-TKA led to significantly inferior implant survival than TKA.

Trying to compare the findings of the current study with those of previous research, it appears that only six studies directly compared UKA-TKA to TKA so far [4, 13, 22, 23, 27, 30]. Among those publications, only Pearse et al. analyzed both the survival rate and a knee score outcome [27]. Similar to the findings of the current study, Pearse et al. reported significantly inferior implant survival of UKA-TKA patients compared to TKA patients (*p* < 0.001). For UKA-TKA, they stated a value of 1.97 for revisions for 100 observed component years, which equals a 10-years survival of approximately 80% compared to a 10-years survival of approximately 95% for the TKA patients.

Regarding the Oxford knee score 6 months postoperatively, UKA-TKA patients of Pearse et al. showed a mean score of 30 compared to a mean score of 37 in the TKA group (*p* < 0.001). The results from that study are in good agreement with the findings of the current study. Except for the fact that the UKA-TKA survival rate of the current study was even lower (10-years survival of 74%). To our best knowledge, the study of Pearse was the only previous study that also analyzed both patient-reported outcome and 10-years implant survival like it was done in our study.

Regarding the other five studies which previously compared UKA-TKA to TKA, none of them investigated survival rates [4, 13, 22, 23, 30]. Miller et al. retrospectively investigated 35 UKA-TKA and 100 TKA and analyzed Knee Society Score and complication rates [22]. The authors reported inferior Knee Society Score among UKA-TKA patients and also a higher complications rate. Also Jarvenpaa et al. conducted a retrospective study on 21 UKA-TKA and 28 TKA patients and collected data on complications, range of motion, WOMAC, VAS pain, and walking distance [13].

For the UKA-TKA patients, the authors reported significantly worse WOMAC pain and WOMAC stiffness, what is in good agreement with the current study.

Becker et al. retrospectively analyzed 28 UKA-TKA and 28 TKA patients [4]. The authors used the Knee Society Score and the WOMAC as patient-reported outcome and additionally also analyzed the range of motion, the insert thickness, and also radiographic parameters. Becker et al. reported significantly thicker inserts, lower range of motion, and worse knee scores among the UKA-TKA patients. The latter fact again being congruent with our findings.

The only conflicting findings come from O’Donnell et al. [23]. The authors compared 55 UKA-TKA with 55 TKA and analyzed the Knee Society Score, the range of motion, and radiographic outcome parameters. In contrast to the findings of the current study, O’Donnell et al. did not identify significant differences between the two groups and hence concluded that the clinical outcome of UKA-TKA is similar to that of TKA.

Sarraf et al. reported on a large population of 374 UKA-TKA and 251,803 TKA but only investigated the height of the insert and the constraint needed [30]. The authors reported significantly higher insert thicknesses in the UKA-TKA group (10 mm vs. 12.8 mm) and also a more frequent demand of implant constraint in the UKA-TKA group (4.19% vs. 2.15%). Due to the absolute incongruence in types of outcome parameters used, that study cannot be compared with the current study. In synopsis of those four previous studies [4, 13, 22, 23] that analyzed only patient-reported outcome (but not survival) in both UKA-TKA and TKA patients, three found worse scores among UKA-TKA patients and one did not. Those findings from previous research are now supported by the findings from the current study.

Regarding reasons for converting a UKA to a TKA (types of UKA failure), the current study found pain, loosening, and progression of osteoarthritis as the major reasons. This is in perfect agreement with the 2021 reports from the arthroplasty registries of the Great Britain and Australia. The following limitations shall be acknowledged.

First, it was a retrospective study with the typical weaknesses associated with such studies: selection bias, information bias, inability to investigate parameters other than those previously collected during clinical routine, reliance on data collected by others etc.

Second, although previously suggested [33], we did not succeed in collecting physical activity data and health-related quality of life data in conjunction with the knee-specific WOMAC data. Third, the types of implants in the UKA-TKA group were heterogenous, both the UKA being explanted and the TKA implants used for revision. However, the latter limitation is true for all previous studies that compared UKA-TKA to TKA [4, 13, 22, 23, 27, 30]. Another limitation of the study is that only those cases from the arthroplasty registry could be included who were previously operated at our institution. The same type of investigation with all cases from the registry would have been more powerful. Furthermore, it is also regarded as limitation that we cannot explain the causality of the fact that UKA-TKA had inferior outcome than TKA. It may be speculated whether this is due to the fact that for a second time a soft tissue approach has to be performed.

It may also be discussed whether the bone loss or medial instability which has to be handled during many UKA-TKA procedures is to blame for the impaired outcome. It should also be acknowledged that we only investigated TKA performed with off the shelf implants, although others suggested that better results may be achieved with more personalized solutions [35]. Finally, the lack of a power analysis is acknowledged.

It is regarded as strengths of the current study that it is the second study so far that investigated both patient-reported outcome (WOMAC) and implant survival over a period of 10 years.

The study findings are regarded as of high clinical relevance. First, the procedure of converting UKA to TKA should not be seen as an easy operation, but should rather be done by surgeons with considerable experience in both primary and revision knee arthroplasty. Second, the inferior outcome of a later conversion of UKA to a TKA should be discussed with a patient already when opting for a UKA.

## Conclusions

Based on our findings it is concluded that patients who received a TKA after UKA have inferior results than those that directly receive a TKA. This is true for both patient-reported knee outcome and prosthesis survival. Converting UKA to TKA should not be seen as an easy operation, but should rather be done by surgeons with considerable experience in both primary and revision knee arthroplasty.
