# Correction to: The clinical outcome of total knee arthroplasty is compromised by a previously implanted medial unicondylar knee arthroplasty

**DOI:** 10.1007/s00402-023-05101-8

**Published:** 2023-10-28

**Authors:** Michael C. Liebensteiner, Alexander Ruzicka, Maximilian Hinz, Hermann Leitner, Alois Harrasser, Dietmar Dammerer, Martin Krismer

**Affiliations:** 1 Department for Orthopaedic Surgery, Medical University Innsbruck, Innsbruck, Austria; 2 Department of Orthopaedic Sports Medicine, Technical University of Munich, Munich, Germany; 3 Department of Clinical Epidemiology, Tyrolean Federal Institute for Integrated Care, Tirol Kliniken GmbH, Innsbruck, Austria; 4 Department of Orthopaedics and Traumatology, University Hospital Krems, Krems, Austria

**Correction to: Archives of Orthopaedic and Trauma Surgery (2023) 143:4331–4337** 10.1007/s00402-023-04829-7

The original version of this article unfortunately contained a mistake. The corrected details are provided below:

All authors’ first names were missing and correct author names should have appeared as below.

- Michael C. Liebensteiner
- Alexander Ruzicka
- Maximilian Hinz
- Hermann Leitner
- Alois Harrasser
- Dietmar Dammerer
- Martin Krismer

